# Serum fibroblast growth factor 21 levels is associated with lower extremity atherosclerotic disease in Chinese female diabetic patients

**DOI:** 10.1186/s12933-015-0190-7

**Published:** 2015-03-11

**Authors:** Xiaoyan Zhang, Yanyun Hu, Hui Zeng, Lianxi Li, Jungong Zhao, Jun Zhao, Fang Liu, Yuqian Bao, Weiping Jia

**Affiliations:** Department of Endocrinology & Metabolism, Shanghai Jiao-Tong University Affiliated Sixth People’s Hospital; Shanghai Clinical Medical Center of Diabetes; Shanghai Key Clinical Center of Metabolic Diseases; Shanghai Institute of Diabetes; Shanghai Key Laboratory of Diabetes, Shanghai, China; Department of Interventional Radiology, Shanghai Jiao-Tong University Affiliated Sixth People’s Hospital, Shanghai, China; Department of Vascular Surgery, Shanghai Jiao-Tong University Affiliated Sixth People’s Hospital, 600 Yishan Road, Shanghai, 200233 China

**Keywords:** Fibroblast growth factor 21, Lower extremity atherosclerotic disease, Type 2 diabetes

## Abstract

**Background:**

Fibroblast growth factor 21 (FGF21) is an emerging metabolic regulator associated with glucose and lipid metabolism, and it is still unclear whether FGF21 is related to atherosclerosis. Here, we explored the potential link between FGF21 and lower extremity atherosclerotic disease (LEAD) in type 2 diabetic patients.

**Methods:**

A cross-sectional study was conducted on 504 type 2 diabetic patients (283 men, 221 women). LEAD was defined by Ankle-brachial index (ABI) <0.9 and lower extremity arterial plaque evaluated by color Doppler ultrasound. Serum FGF21 concentrations were quantified by a sandwich enzyme-linked immunosorbent assay.

**Results:**

The total FGF21 levels of male and female patients had no significant differenence ((299.14(177.31-534.49) vs 362.50(214.01-578.73), *P*=0.516). Serum FGF21 levels in LEAD group were significantly higher than non-LEAD group in females (385.34(243.89-661.54) vs 313.13(156.38-485.79), *P*=0.006), while not in male patients (295.52(177.09-549.64) vs 342.09 (198.70-549.87), P=0.613). In diabetic women, subjects with LEAD had significantly higher serum FGF21 regardless of non-alcoholic fatty liver disease (NAFLD) (*P* < 0.05). And serum FGF21 levels were positively correlated with waist circumference and systolic blood pressure after adjusted for age and BMI (*r*=0.198, *P*=0.004; *r*=0.152, *P*=0.027; respectively). Moreover, FGF21 was independently tied to femoral intima-media thickness (FIMT) (β=0.208, *P*=0.031). After adjusted for other LEAD risk factors, FGF21 was demonstrated to be an independent risk factor for LEAD in type 2 diabetic women (OR, 1.106; 95%CI 1.008-1.223; *P*=0.028). In addition, FGF21 was negatively correlated with estradiol in premenopausal diabetic women (*r*=−0.368, *P*=0.009). After adjusted for estradiol, serum FGF21 levels were still positively associated with FIMT in premenopausal diabetic women (r=0.381, *P*=0.007). In diabetic men, serum FGF21 levels were correlated with triglyceride and C-reactive protein even after adjusted for age and BMI (*r*=0.204, *P*=0.001; *r*=0.312, *P* < 0.001; respectively). However, serum FGF21 was not an independent impact factor for LEAD in men (*P* > 0.05).

**Conclusions:**

Serum FGF21 level independently and positively links LEAD in Chinese women with type 2 diabetes. The gender difference may be due to different estrogen levels.

## Background

Atherosclerosis is a progressive disease which affects multiple vascular beds. And its clinical consequence including coronary arterial disease, cerebrovascular disease and peripheral artery disease (PAD) are potentially life-threatening. Even the patients with higher subclinical atherosclerosis risk have significantly higher cumulative incidence rate of cardiovascular events [1]. In diabetic patients, the onset of atherosclerosis was earlier [2]. Diabetics had significantly higher pulse wave velocity, increased carotid intima-medial thickness and more plagues than non-diabetes [3]. In fact, atherosclerotic lesions were more frequent in femoral arteries than carotid arteries independent of increasing number of risk factors [4]. As one of common diabetic macrovascular complications, lower extremity atherosclerotic disease (LEAD) or diabetic PAD of lower extremity, was one of the major causes of foot ulceration and amputation [5]. Early detection and treatment of LEAD is critical to prevent amputation and mortality of diabetic population. Despite the fact that LEAD is an independent predictor of cardiovascular and cerebrovascular ischemic events, this particular manifestation of systemic atherosclerosis is largely under-diagnosed and undertreated [6]. Therefore, it is vital for diabetic patients to recognize lower limb atherosclerosis and control its risk factors as early as possible.

Fibroblast growth factors and their receptors have a wide range of biological functions. As we all known, basic fibroblast growth factor (bFGF), one of FGFs isomer, is involved in atherosclerosis formation [7]. But as a member of the FGFs subfamily, fibroblast growth factor 21 (FGF21) plays an important role in regulating glucose and lipid metabolism and insulin sensitivity in animals. Pharmacological doses of FGF21 produce anti-diabetic, lipid-lowering, and weight-reducing effects in rodents. And mice with over-expression of FGF21 were protected from diet-induced obesity [8], while FGF21 knockout mice developed mild obesity and impaired glucose homeostasis as these mice became aged [9]. Gaich et al. reported the first clinical trial that FGF21 analog improved the lipid profile of obese subjects with type 2 diabetes [10]. And FGF21 may be a promising therapeutic target in obesity-related diseases [11]. Actually, despite of FGF21 reduction in type 1 diabetes and latent autoimmune diabetes in adults (LADA) [12], circulating FGF21 levels were elevated in obesity [13], type 2 diabetes [14], dyslipidemia [15] and non-alcoholic fatty liver disease (NAFLD) [16]. Shen Y et al. also showed that FGF21 concentrations increased in coronary heart disease [17] and “FGF21 resistance”, a phenomenon reminiscent of hyperinsulinemia and insulin resistance might be one of the reasons for the increase of elevated FGF21 [18].

Except for these above findings that FGF21 was associated with metabolic dysfunction and the well-established link between metabolic disorders and cardiovascular disease, few clinical studies have reported the potential connection between FGF21 and atherosclerosis especially LEAD. An SY et al. found that subjects with carotid artery plaque had higher serum FGF21 levels than those without complications [19]. A study from Ulu SM et al. indicated that FGF21 was an independent determinant of arterial stiffness in patients on dialysis [20]. Thus, the aim of the present study was to clarify the possible link between serum FGF21 levels and LEAD in diabetes patients.

## Research design and methods

### Study population

Consecutive 504 type 2 diabetic inpatients at the Shanghai Clinical Medical Center of Diabetes from January 2013 to December 2013 were enrolled in the study. They were mainly local from 16 districts of Shanghai and were admitted for uncontrolled hyperglycemia or diabetic complications. The diagnostic criteria of diabetes was based on the American Diabetes Association standards [21]. Patients with type 1 diabetes, other specific types of diabetes or acute complications of diabetes were excluded. All the enrolled patients continued their previous glycemic control regimen including hypoglycemic drugs and (or) insulin. The study was approved by the Ethics Committee of the Shanghai Jiao-Tong University Affiliated Sixth People’s Hospital. The informed consents were completed by all the participants, which were abided by the principle of the Declaration of Helsinki.

### Data collection

All subjects completed a questionnaire that collected general background information including present and previous illness, medication, alcohol consumption and smoking status. Hypertension was defined as systolic blood pressure (SBP) ≧140 mmHg or diastolic blood pressure (DBP) ≧90 mmHg or history of antihypertensive medicine administration. Height, weight, waist circumference (W) and blood pressure were assessed on a standardized form by the same physician during the health check-up. Body mass index (BMI) was calculated as body weight (in kg) divided by the square of the height (in m). All the patients had an overnight fast prior to the blood samples collection.

### Laboratory measurements

Blood samples were transported to the laboratory of Shanghai Clinical Medical Center of Diabetes as needed after collected. Fasting plasma glucose (FPG) and 2-hour postprandial plasma glucose (2hPG) were measured by glucose oxidase method. Glycosylated hemoglobin A1c (HbA1c) was determined by high-pressure liquid chromatography and glycated serum albumin (GA) was measured by the liquid enzymatic assay. Serum alanine aminotransferase (ALT) and serum lipids including total cholesterol (TC), triglyceride (TG), highdensity lipoprotein cholesterol (HDL-C), low density lipoprotein cholesterol (LDL-C) were performed by enzymatic method. The glomerular filtration rate (GFR) was determined by technetium-99 m diethyl triamine penta-acetic acid (Tc^99m^-DTPA) clearance. Serum C-reactive protein (CRP) was measured by particle-enhanced immunonephelometric assay (Dade Behring Inc., Newark, NJ, USA). The serum sex hormone including testosterone (T), estradiol (E2), progesterone (P), luteinizing hormone (LH), follicle stimulating hormone(FSH), prolactin (PRL), dehydroepiandrosterone sulfate(DHEA-S) were tested by Chemoluminescence (Diasorin company, Italy). Serum FGF21 concentration was determined by enzyme-linked immunosorbent assay (ELISA) (Antibody and Immunoassay services, University of Hong Kong), which give intra-batch and inter-batch variations were 7.8% and 9.1%, respectively. Serial dilutions of recombinant FGF21 were included in all assays as a standard. Duplicate measurements were obtained for all samples.

### ABI and Ultrasonography measurements

American Nieolet Versalab duplex doppler blood flow detector was used to determine brachial and ankle arterial pressure. Patients supine, with 12 cm × 40 cm gas sleeve respectively in bilateral ankle and upper arm, with doppler stethoscope to assist the acquisition dorsalis pedis or tibial artery, posterior tibial artery and brachial artery systolic blood pressure. Ankle-brachial index (ABI) was calculated by the higher SBP in the dorsalis pedis and posterior tibial artery devided by the higher brachial SBP. The lower value of ABI in either limb was used for analysis. The arterial lesion of lower extremity artery were evaluated by color Doppler ultrasound examination, and the femoral intima-media thickness (FIMT) were also recorded. Color duplex ultrasonography was conducted by three trained, certified sonographers using a Acuson Sequoia 512 scanner (Siemens Medical Solutions, Mountain View, CA) with a 5–13 MHz linear transducer according to our previous method [22]. The study procedure involved scanning bilateral common femoral artery, profunda femoris artery, superficial femoral artery, popliteal artery, anterior tibial artery, posterior tibial artery, and peroneal artery for the presence of atherosclerotic plaque and stenosis. The FIMT on both sides was measured as the distance between the leading edge of the lumen-intima echo and the leading edge of the media-adventitia echo. Mean FIMT was defined as the mean values of bilateral FIMTs. Lower limb atherosclerotic plaque was defined as the presence of a focal structure encroaching into the arterial lumen of 0.5 mm or at least 50% greater than the thickness of the surrounding vessel wall or IMT of >1.5 mm in any of the above-mentioned lower limb arteries segments based on the Mannheim consensus [23]. LEAD was defined by ABI < 0.9 and lower extremity arterial plaque existed. Those with an ABI > 1.3 were excluded from the analysis to avoid those with significant medical artery layer calcification, which is independent of atherosclerotic plagues. Others individuals were named as non-LEAD.

### Diagnostic criteria for non-alcoholic fatty liver disease(NALFD)

NALFD was diagnosed by B ultrasonography. Hepatic steatosis was defined by a diffuse increase of fine echoes in the liver parenchyma compared with that in the kidney or spleen parenchyma according to the 2010 Prevention and Treatment Guidelines for NALFD published by the society of Hepatology, Chinese Medical Association [24].

### Data analysis

All the statistical analysis was performed by SPSS 21.0 (SPSS Inc., Chicago, IL). The one-sample Kolmogorov-Smirnov test was performed to determine normality of the data distribution. Normally distributed data were expressed as mean ± standard deviation (SD), and data with skewed distribution were expressed as median with interquartile range. Differences between groups were evaluated with Student’s *t* test or Mann–Whitney *U* test. Categorical variables were presented as frequency percentage, and intergroup comparisons were analyzed using a Chi-square test. The association between FGF21 and other variables were evaluated with Spearman correlation and partial correlation analysis. Logistic regression analysis was performed to evaluate the odds ratio of LEAD. Multiple stepwise regression analysis was used to explore the influence of different variables on FIMT. To determine the independent predictors for the presence of LEAD, all the conventional risk factors related with LEAD as well as the disease-related therapies were tested in multivariable logistic regression. The threshold of statistical significance was set at 0.05 for two-tailed P-values.

## Results

**Figure 1:**
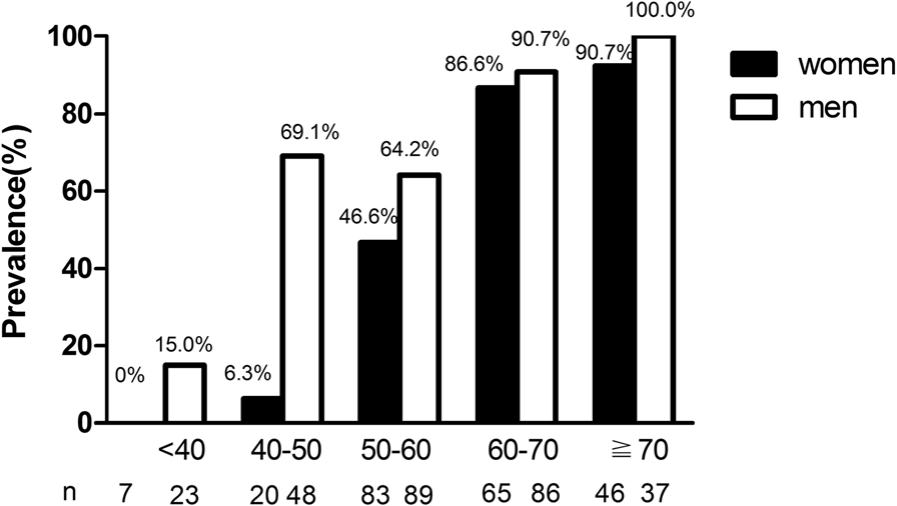
**Comparison of prevalence of LEAD stratified by age and sex in type 2 diabetic patients.** White bars: men; black bars: women. Trend analysis, *p* < 0.001.

The mean age of the 504 study subjects was 58 years, the media diabetes duration was 9 years, and the median level of serum FGF21 was 327.03 ng/mL, with an interquartile range of 190.05–545.55 ng/mL. Comparison of the prevalence of LEAD stratified by sex and age was shown in Figure 1. The prevalence of LEAD significantly increased with age both in diabetic men and women (*P* < 0.05). FGF21 levels were not significantly different between male and female patients (299.14(177.31-534.49) *vs* 362.50 (214.01-578.73), *P* = 0.516).

**Figure 2:**
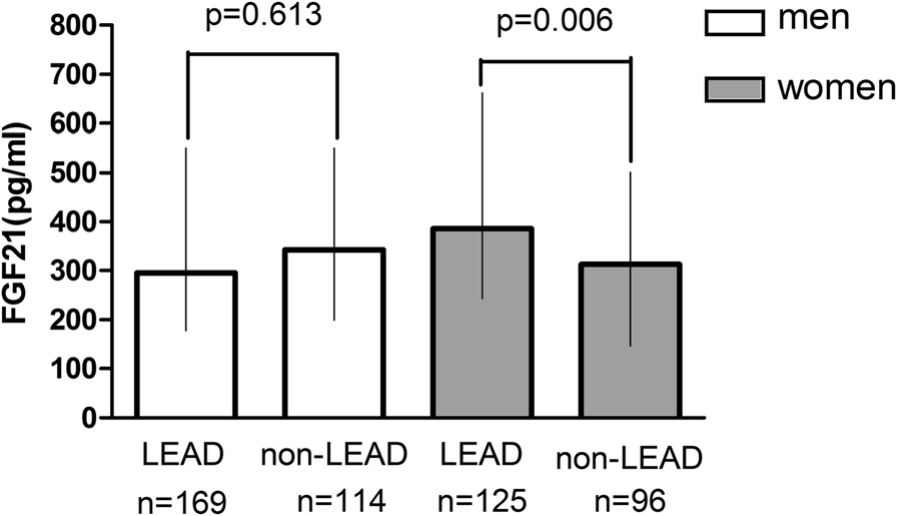
**Comparison of serum FGF21 levels in LEAD and non-LEAD subgroup stratified by sex.** White bars: men; Grey bars: women.

**Figure 3:**
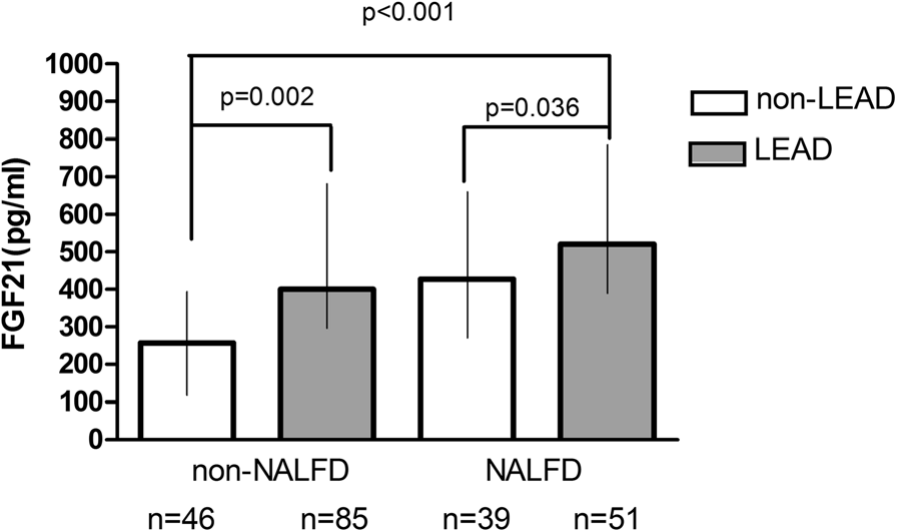
**Serum FGF21 levels among subjects with NAFLD and/or LEAD (data from women only).** White bars: non-LEAD; Grey bars: LEAD.

The clinical characteristics of LEAD and non-LEAD patients with respect to sex are shown in Table 1. In female subgroup, there were significant differences in age, diabetes duration, SBP, prevalence of hypertension and anti-hypertensive therapy between LEAD and non-LEAD patients (*P <* 0.05). In male subgroup, there were significant differences in age, diabetes duration, prevalence of hypertension and anti-hypertensive therapy, FPG, TC, TG, LDL-C, HbA1c and GA between LEAD and non-LEAD patients (*P <* 0.05). FIMT values were significantly higher in LEAD than in non-LEAD group in both male patients and female patients (*P <* 0.05). Serum levels of FGF21 in LEAD group were significantly higher than non-LEAD group in female patients (385.34(243.89-661.54) vs. 313.13(156.38-485.79), *P* = 0.006), while not in male patients (295.52(177.09-549.64) *vs.* 342.09(198.70-549.87), *P* = 0.613) (Figure 2). As is shown in Figure 3, both subjects with and without NAFLD showed a significant elevation of serum FGF21 levels in the LEAD group compared to the non-LEAD group (*P <* 0.05).

**Table 1:** **Clinical and biochemical characteristics of participants**

**Variables**	**Men**		***P***	**Women**		***P***
	**LEAD**	**Non-LEAD**		**LEAD**	**Non-LEAD**	
Age(year)	61.75 ± 10.31	47.55 ± 10.07	<0.001	64.40 ± 9.79	56.62 ± 9.52	<0.001
Diabetes duration(year)	10(5–15)	3(0.48-10)	<0.001	13.14 ± 7.73	7.85 ± 5.64	<0.001
Body mass index(kg/cm^2^)	23.59 ± 3.53	25.99 ± 3.20	0.194	25.12 ± 3.83	25.37 ± 3.89	0.655
Waist circumference(cm)	93.23 ± 10.89	94.80 ± 10.17	0.273	91.19 ± 11.35	88.02 ± 10.49	0.055
Systolic blood pressure(mmHg)	130(120–140)	130(120–140)	0.503	135(123.5-150)	129(120–135)	<0.001
Diastolic blood pressure(mmHg)	80(70–85)	80(78–90)	0.010	80(75–85)	80(70–85)	0.268
Fasting plasma glucose(mmol/L)	6.88(5.92-9.07)	7.92(6.50-10.36)	0.007	8.03 ± 2.62	2.97 ± 0.91	0.409
2 h postprandial plasma glucose(mmol/L)	12.96 ± 4.40	13.73 ± 4.53	0.212	14.31 ± 4.57	13.48 ± 4.61	0.232
Fasting C-peptide(ng/ml)	1.93 ± 1.03	2.12 ± 1.13	0.213	1.95 ± 1.07	2.07 ± 1.15	0.472
2-h postprandial C peptide(ng/ml)	4.98 ± 3.18	5.07 ± 3.50	0.854	4.90 ± 3.26	5.11 ± 2.99	0.664
Total cholesterol(mmol/L)	4.46 ± 1.11	4.98 ± 1.17	0.001	4.92 ± 1.10	5.08 ± 1.16	0.352
Triglyceride(mmol/L)	1.92(1.16-3.63)	1.40(0.93-2.10)	0.005	1.42(1.05-1.93)	1.46(0.98-2)	0.720
High-density lipoprotein cholesterol(mmol/L)	1.00 ± 0.24	0.97 ± 0.21	0.397	1.15 ± 0.32	1.18 ± 0.33	0.481
Low-density lipoprotein cholesterol(mmol/L)	2.63 ± 0.89	2.96 ± 0.80	0.006	2.86 ± 0.97	2.97 ± 0.91	0.409
Glycated hemoglobin A1c(%)	8.0(7.1-9.85)	9.1(7.45-10.85)	0.023	8.82 ± 1.86	8.62 ± 1.85	0.459
Glycated serum albumin(%)	22.30 ± 7.18	24.44 ± 8.07	0.044	20.9(17.75-26.30)	20.10(17.20-25.3)	0.364
Alanine aminotransferase(U/L)	21(14–27)	24(18–36.5)	0.065	18(13–31.75)	22(14.75-30)	0.303
Aspartate aminotransferase(U/L)	19(16–23)	19(15–28)	0.423	19(16–24)	20(15–25.5)	0.894
γ-glutamyl transpeptidase(U/L)	27(19–39)	31(21–42.5)	0.065	23(16.75-39)	28(18.50-44)	0.160
Glomerular filtration rate(ml/min/1.73 m^2^)	97.04 ± 24.04	96.34 ± 24.92	0.845	94.67 ± 25.79	98.37 ± 23.23	0.355
C reactive protein(mg/L)	1.67(0.64-2.57)	1.10(0.56-2.35)	0.347	1.3(0.53-2.65)	1.45(0.75-3.11)	0.256
Femoral intima-media thickness(mm)	0.85 ± 0.06	0.67 ± 0.12	<0.001	0.86 ± 0.03	0.70 ± 0.11	<0.001
Smoking(%)	55.8	54.9	0.888	0.8	2.7	0.278
Drinking(%)	24.5	19.5	0.233	1.6	1.4	0.903
Hypertension(%)	60.7	36.6	<0.001	62.7	37.0	<0.001
Anti-hypertensive therapy(%)	47.1	18.9	<0.001	48.6	20.3	<0.001
Anti-diabetic therapy(%)	78.9	77.1	0.732	68.9	66.4	0.453
Lipid-lowing therapy(%)	15.8	14.3	0.677	12.8	10.3	0.566
Estradiol	115.24 ± 58.72	116.54 ± 56.01	0.889	70.94(38.73-104.91)	88.95(50.10-165.27)	0.039
Testosterone	12.61 ± 5.25	12.64 ± 4.56	0.972	1.18(0.89-1.55)	1.26(0.94-1.69)	0.610
Progestogen	1.04(0.75-1.47)	1.03(0.71-1.33)	0.516	0.82(0.65-1.42)	0.80(0.64-1.90)	0.756
Follicle-Stimulating Hormone	9.43(6.40-14.41)	6.50(4.00-9.04)	<0.001	52.53 ± 23.61	38.08 ± 25.33	0.001
Luteinizing hormone	5.92(3.98-9.56)	4.96(3.24-6.48)	0.002	23.78 ± 10.53	20.16 ± 13.69	0.960
Prolactin	168.83(136.86-214.16)	171.39(109.74-242.44)	0.002	193.70 ± 85.48	185.66 ± 138.83	0.707
Dehydroepiandrosterone sulfate	201.13 ± 106.74	260.10 ± 117.77	0.001	126.50 ± 66.74	173.73 ± 81.99	<0.001

In order to find out the influencing factors for FGF21, Spearman correlation analysis of clinical and biochemical parameters with FGF21 were undertaken. In female patients, FGF21 was positively correlated with age, BMI, W, SBP, DBP, TG, FCP and 2hCP, but was negatively associated with HDL-C (*P* < 0.05). Serum FGF21 level was not correlated with CRP in female patients (*P >* 0.05). Even after adjusted for age and BMI, FGF21 was still positively associated with W and SBP (r = 0.198, *P* = 0.004; *r* = 0.152, *P* = 0.027; respectively). In male group, serum FGF21 level was correlated with TG and CRP (*P* < 0.05) (Table 2). After adjusted for age and BMI, FGF21 level was still correlated with TG and CRP (r = 0.204, P = 0.001; r = 0.312, *P* < 0.001; respectively).

**Table 2:** **Correlation of FGF21 with anthropometric and biochemical variables**

**Covariables**	**Women**		**Men**	
	**r**	***P***	***r***	***P***
Age(year)	0.031	0.647	−0.011	0.858
Diabetes duration(year)	−0.030	0.662	−0.015	0.805
Body mass index(kg/cm^2^)	0.160	0.018	0.081	0.179
Waist circumference(cm)	0.188	0.005	0.074	0.218
Systolic blood pressure(mmHg)	0.200	0.003	−0.036	0.551
Diastolic blood pressure(mmHg)	0.176	0.009	−0.036	0.557
Fasting plasma glucose(mmol/L)	0.005	0.945	−0.003	0.958
2 h postprandial plasma glucose(mmol/L)	0.073	0.290	−0.092	0.139
Fasting C-peptide(ng/ml)	0.168	0.017	0.102	0.106
2-h postprandial C peptide(ng/ml)	0.149	0.034	0.014	0.845
Total cholesterol(mmol/L)	0.036	0.606	0.088	0.160
Triglyceride(mmol/L)	0.245	<0.001	0.21	0.001
High-density lipoprotein cholesterol(mmol/L)	−0.192	0.006	−0.091	0.147
Low-density lipoprotein cholesterol(mmol/L)	−0.044	0.524	−0.007	0.906
Glycated hemoglobin A1c(%)	0.047	0.493	0.057	0.366
Glycated serum albumin(%)	−0.001	0.985	−0.011	0.865
C reactive protein(mg/L)	0.150	0.032	0.222	<0.001
Femoral intima-media thickness(mm)	0.151	0.048	0.022	0.709

Since there was gender difference of association between FGF21 and LEAD, women were further divided into two subgroups and compared according whether menopause or not. There was no significant difference in FGF21 levels between menopausal and pre-menopausal women [360.51(228.16-529.75) vs 363.54(208.57-593.95), P > 0.05]. It showed that FGF21 was negatively correlated with E2 in premenopausal diabetic women (*r* = −0.368, *P* = 0.009) (Table 3). After adjusted for estradiol, serum FGF21 level was still positively associated with FIMT in premenopausal diabetic women (r = 0.381, P = 0.007).

**Table 3:** **Correlation of FGF21 with sex hormone by Spearman analysis**

**Covariables**	**Postmenopausal (n = 143)**		**Premenopausal (n = 78)**		**Men (n = 283)**	
	**r**	***P***	**r**	***P***	**r**	***P***
Estradiol	0.07	0.593	−0.368	0.009	0.092	0.212
Testosterone	0.029	0.795	0.149	0.288	−0.09	0.213
Progestogen	−0.221	0.201	−0.148	0.383	0.045	0.602
Follicle-Stimulating Hormone	−0.029	−0.798	0.009	0.949	−0.093	0.2
Luteinizing hormone	0.034	0.762	−0.104	0.456	0.003	0.966
Prolactin	−0.033	0.769	−0.147	0.288	0.113	0.122
Dehydroepiandrosterone sulfate	−0.076	0.493	0.212	0.123	−0.018	0.805

In order to determine which factors were independently associated with LEAD, logistic regression was performed. Independent variables were set as metabolic risk factors (including age, diabetes duration, smoking status, presence of hypertension,ALT, GFR, HbA1c, waist circumference, dyslipidemia, anti-diabetic therapy, anti-hypertensives, lipid-lowering therapy and serum FGF21 levels). In female patients, logistic regression analysis of LEAD showed that age (OR, 1.235; 95%CI (1.133-1.347); P < 0.001), hypertension (OR, 3.231; 95%CI 1.102-9.470; P = 0.033), FGF21 (OR, 1.106; 95%CI 1.008-1.223; P = 0.028) were independent impact factors for LEAD. In male patients, only age (OR, 1.171; 95%CI (1.097-1.249); *P* < 0.001) was independent associated factor for LEAD (Table 4). After adjustment for the confounding variables described above, multiple stepwise regression analysis showed that the age (*β* = 0.519, *P* < 0.001), FGF21 (*β* = 0.208, *P* = 0.031), HbA1c (*β* = 0.225, *P* = 0.020) were independent risk factors for FIMT in type 2 diabetes women. While in male patients, only age (*β* = 0.539, *P* < 0.001) was independently associated with FIMT (Table 5).

**Table 4:** **Independent factors for LEAD in men and women by multivariable logistic regression analysis**

	**β**	**S.E**	**Wald**	**OR(95% CI)**	***P***
Female					
Age	0.211	0.044	22.868	1.235(1.133-1.347)	<0.001
Hypertension	1.173	0.549	4.570	3.231(1.102-9.470)	0.033
FGF21	0.113	0.036	3.537	1.106(1.008-1.223)	0.028
Male					
Age	0.157	0.033	22.719	1.171(1.097-1.249)	<0.001

**Notation:** S.E., standard error; OR, odds ratio; CI, confidence interval. Variables included in the model were age, diabetes duration, smoking status, presence of hypertension, ALT, GFR, HbA1c, W, dyslipidemia, anti-diabetic therapy, anti-hypertensives, lipid-lowering therapy and serum FGF21 levels.

**Table 5:** **Multiple stepwise linear regression analysis of FIMT**

	**Standardized β**	**t**	***P***
Female			
Age	0.519	5.495	<0.001
FGF21	0.208	2.202	0.031
HbA1c	0.225	2.387	0.020
Male			
Age	0.539	0.254	<0.001

**Notation:** Variables included in the model were age, diabetes duration, smoking status, presence of hypertension, ALT, GFR, HbA1c, W, dyslipidemia, anti-diabetic therapy, anti-hypertensives, lipid-lowering therapy and serum FGF21 levels.

## Discussions

In this study we provide the evidence for the first time that elevated serum FGF21 levels are associated with LEAD in female type 2 diabetic patients independent of established risk factors.

Our research group had shown that increased level of serum FGF21 was associated with NAFLD and mRNA expression of FGF21 has been shown to increase in hepatic biopsies [16]. Furthermore, a 3 year follow-up of NALFD subject outcome indicated that serum FGF21 level might be a clinically-relevant disease biomarker for NALFD [25]. In the current study, a significant elevation of serum FGF21 among LEAD subjects was found independently of NALFD status. Multivariable logistic regression analysis also identified serum FGF21 level as one of the independent risk factors for LEAD.

The mechanism linking FGF21 with atherosclerosis was currently not well understood. Elevated mRNA expression of FGF21 was found in rat cardiac micro-vascular endothelial cells (CMECs) cultured in atherosclerosis-like conditions [26]. Furthermore, exogenous FGF21 infusion to the CMECs atherosclerosis promoting culture significantly inhibited the apoptosis of cells. These findings suggested that up-regulated FGF21 expression might be protective at the early stage of atherosclerosis, helping the cells to recover normal endothelial function. FGF21 also has antioxidant effects in atherosclerotic rat, such that increased levels of superoxide dismutase, reduced glutathione, and reduced malondialdehyde [27]. Another study found the protective effect of FGF21 on atherosclerosis might be in part due to its inhibition on endoplasmic reticulum stress-mediated apoptosis [28].

Actually in human study, circulating FGF21 levels are elevated in obesity, type 2 diabetes and dyslipidemia. It was proposed that the elevated level of FGF21 was attributed to FGF21 resistance, a phenomenon reminiscent of hyperinsulinemia and insulin resistance. One of the reasons of elevated FGF21 could be the presence of compensatory response to higher metabolic stress. A recent study suggested that adipose tissue inflammation in obesity could lead to the repression of beta-Klotho expression by TNF alpha and impaired FGF21 in adipocytes [29]. Hence we can infer it is very likely that similar actions lead to the FGF21 resistance in subclinical inflammation such as LEAD. Thus, it is possible that the elevated FGF21 observed in the LEAD subjects of our study represent a similar compensatory mechanism, by which the system is attempting to protect against atherosclerosis.

In our study, we observed the gender-specific association between serum FGF21 and LEAD. Chow WS et al. also found this association was gender-specific, they found that serum FGF21 levels positively correlated with carotid IMT in women (r = 0.32; P < 0.001) but not in men (r = 0.06; P = 0.305) [30]. As we all know, E2 protected premenopausal women from cardiovascular disease. While in postmenopausal women, the prevalence of macrovascular diseases was higher. Based on these observations, the relationship between FGF21 and hormonal parameters were assessed in our study. In our data, it was found that FGF21 was negatively correlated with E2 in diabetic women. In polycystic ovary syndrome and healthy subjects, a positive correlation was also found between FGF21 and LH and T (r = 0.43 p = 0.007; r = 0.38, P = 0.02, respectively) [31]. Another study found there was a significantly negative correlation between FGF21 and dehydroepiandrosterone sulfte (DHEAS) (r = −0.309 p = 0.003) [32]. This significant correlation between FGF21 and sex hormone in our study group arises a need of new studies to explain the potential role of FGF21 in atherosclerosis pathogenesis. Our finding of the gender-specific association between serum FGF21 and LEAD remains to be confirmed in further studies.

In our study, FGF21 was found to positively correlated to BMI, W, TG, but negatively link with HDL-C in diabetic patients. Some of these relationships have been described in the previous studies [13,33,34]. But there are only limited information about the relationships of FGF21 and hypertension in literature. In our study, positive associations of FGF21 and SBP were found in diabetic patients. The same result was found in Japanese subjects [35]. CRP reflecting systemic inflammation, was a well-established marker of atherosclerosis and one of the classical biomarkers for increasing risk of PAD [36]. And CRP might play a role in the progression of PAD in diabetic patients [37]. A recent study of 69 newly diagnosed diabetes subjects demonstrated a positive correlation between serum FGF21 levels and CRP [38]. Similarly, serum FGF21 levels also linked with CRP in the present study (*r* = 0.15 *P* = 0.032).

Consistent with other studies, we found that some of the traditional risk factors for atherosclerosis were also present in this population. As expected, age, hypertension were independently associated with the presence of LEAD in the type 2 diabetes women. Therefore, strict control of hypertension is important in order to prevent atherosclerosis in the lower limb arteries in diabetic patients.

The strength of this study is that GFR was directly measured by the ^99m^Tc-DTPA renal dynamic imaging rather than estimated from serum creatinine, which was more accurate and avoided the impact from serum creatinine. Several previous studies reported a close association between chronic renal insufficiency and PAD [39]. Our previous study also revealed that the degree of peripheral arterial lesion was significantly correlated with renal function and GFR. In our study, the difference of GFR between LEAD and Non-LEAD women patients was not significant, the increased serum FGF21 did not result in the dysfunction of kidney.

Lenart-Lipińska M et al. showed that serum FGF21 is predictive of combined cardiovascular morbidity and mortality in patients with type 2 diabetes at 24 months follow-up [40]. Another larger scale study showed higher baseline plasma FGF21 levels were associated with higher risk of cardiovascular events in patients with type 2 diabetes over 5 years follow-up in 9,697 individuals with type 2 diabetes participating in the Fenofibrate Intervention and Event Lowering in Diabetes (FIELD) study [41]. A follow-up of the outcome in LEAD subjects is necessary to elucidate whether serum FGF21 level might be a clinically-relevant vascular disease biomarker.

### Limitations

There were some limitations of our study. Firstly, the cross-sectional design restricted our ability to assess the evolutionary process of atherosclerotic lesions. Secondly, this was merely a single-center study with a relatively small number of patients. Thirdly, some other confounding affecting factors of LEAD were not excluded. Further *in vivo* and *in vitro* studies are needed to elucidate the essential relationship between FGF21 and LEAD and the underlying detailed mechanism in diabetes.

## Conclusion

In conclusion, serum FGF21 levels independently and positively connect with LEAD in Chinese women with type 2 diabetes after adjusted for the traditional risk factors, and its gender difference may be due to the difference of estrogen levels. Further studies revealing the immanent connection of FGF21 with the pathology of diabetic peripheral vascular disorders may provide a new prospective strategy for LEAD.

## Abbreviations

BMI: Body mass index
W: Waistcircumference
ALT: Alanine aminotransferase
AST: Aspartate aminotransferase
γ-GT: γ-glutamyl transpeptidase
GFR: Glomerular filtration rate
FPG: Fasting plasma glucose
2hPG: 2 h postprandial plasma glucose
HbA1c: Glycated hemoglobin A1c
GA: Glycated serum albumin(%)
FCP: Fasting C-peptide
2hCP: 2-h postprandial C peptide
TC: Total cholesterol
TG: Triglyceride
HDL-C: High-density lipoprotein cholesterol
LDL-C: Low-density lipoprotein cholesterol
